# *Plasmodium falciparum* *dhps* and *dhfr* markers of resistance to sulfadoxine–pyrimethamine five years (2016–2020) after the implementation of seasonal malaria chemoprevention in Cameroon

**DOI:** 10.12688/wellcomeopenres.22347.1

**Published:** 2024-06-20

**Authors:** Pacome V. K. Tchuenkam, Lesley N. Ngum, Innocent M. Ali, Jean Paul K. Chedjou, Akindeh M. Nji, Palmer M. Netongo, Randolph Ngwafor, Peter Thelma N. Niba, Calvino F. Tah, William D. Nana, Germaine Ekoyol, Jude D. Bigoga, Dorothy F. Ashu, Christopher B. Tume, Wilfried F. Mbacham

**Affiliations:** 1 Department of Biochemistry, Faculty of Science, University of Dschang, Dschang, West region, Cameroon; 2 MARCAD Program, The biotechnology Centre, University of Yaounde I, Yaounde, Centre, P 0 Box 8094, Cameroon; 3 Department of Biochemistry, Faculty of Science, University of Yaounde I, Yaounde, Centre, Cameroon; 4 Institute of Medical Research and Medicinal Plants Studies, IMPM/MINRESI, Yaoundé, Centre, BP 13033, Cameroon; 5 Department of Biochemistry, University of Buea, Buea, Southwest, P O box 63, Cameroon; 6 National Malaria Control Program, Ministry of Public Health, Yaoundé, Centre, BP 14386, Cameroon; 7 Department of Biochemistry, Faculty of Science, Universof Bamenda, Bamenda, Northwest, P.O box 39, Cameroon; 8 Centre for Health Implementation and Translation Research, The Fobang Institute, Yaoundé, Centre, BP 8094, Cameroon

**Keywords:** sulfadoxine–pyrimethamine, drug resistance, plasmodium falciparum, chemoprevention, Pfdhps, Cameroon

## Abstract

**Background:**

Antimalarial drug resistance is a major challenge in the fight against malaria. Cameroon implemented seasonal malaria chemoprevention (SMC) with sulfadoxine–pyrimethamine and amodiaquine (SPAQ) to over 1.5 million children aged 3–59 months from 2016, raising concerns whether drug pressure may lead to a selection of known parasite resistance mutations. This study aimed at assessing the profiles of plasmodium falciparum dihydrofolate reductase (DHFR) and plasmodium falciparum dihydropteroate synthase (DHPS) gene mutations that encode enzyme targeting SP before and 5 years after the introduction of SMC in the northern part of Cameroon.

**Methods:**

Dried blood spots were prepared from symptomatic *P. falciparum-*positive children prior to SPAQ administration in 2016 and after the SMC round of 2020. DNA was extracted using the Chelex-100 method, and *dhfr* and *dhps* mutations were determined after a nested polymerase chain reaction–restriction fragment length polymorphism (PCR-RFLP) technique and agarose gel electrophoresis.

**Results:**

405 children with acute uncomplicated malaria were recruited. Of 405 samples, 201/405 (49.63%) were collected in 2016 and 204/405 (50.37%) were collected in 2020. High levels of mutant alleles S108N, C59R, N51I of *dhfr* were obtained both in 2016 and 2020 (174 (100%), 166 (95.4%), 131 (75.3%)); (140 (99.4%), 131 (92.2%), 114 (80.3%)) while the frequency of *dhps* mutant alleles in the A437G and K540E loci stood at 93 (51.9%) and 6 (3.4%) in 2016 and 73 (52.5%) and 4 (2.8%) in 2020, respectively. The quintuple resistant haplotype IRNGE was found in two (1.1%) and one (0.7%) in 2016 and 2020, respectively. No significant difference was observed in the frequency of the studied mutations between the two time points, although we noted a rise in the resistance conferring haplotype IRNG in 2020.

**Conclusions:**

Continuous monitoring is recommended to preempt the widespread occurrence of high-grade resistance bearing parasites in the northern regions of Cameroon.

## Plain Language Summary

Despite all the efforts to fight against the malaria burden, it continues to be a serious threat to people's life. One of the biggest threats to the elimination and eradication of malaria is the development of resistance to known and established drugs used in the treatment and the prevention of malaria. These resistance markers accounts as a result of mutations on some Plasmodium genes. In this study, we accessed the prevalence of key resistance markers of two genes in a region where a prevention strategy (the Seasonal Malaria Chemoprevention) has been implemented for 5 years. More than 400 (201 in 2016 and 204 in 2020) patients were screened for malaria and blood samples collected to access these markers. High levels of these resistance markers were observed both in 2016 and in 2020. We found a slight increase in the resistance markers from 2016 to 2020 though it was not significant. These results raise the need to continue to closely monitor patterns of resistance.

## Introduction

Malaria is still an important threat to global health as it remains one of the most dangerous parasitic diseases impacting the living standards and life expectancy of people in endemic countries ^1– 3^. Although significant progress has been made in the last decade, the disease remains prevalent, especially in Africa. For example, of the 85 endemic countries in the world, the WHO African region is home to about 95% of the global malaria cases ^4^. In Cameroon, approximately 28% morbidity and 18.3% mortality is caused by malaria, with children under the age of 5 responsible for the highest proportion, especially in the North Region and the Far North Region ^5, 6^. The transmission of malaria is intense and seasonal in the northern regions of Cameroon when compared with the southern regions, where it is perennial. In 2016, the government of Cameroon started the implementation of seasonal malaria chemoprevention (SMC) in the North Region and Far North Region ^7, 8^. This prevention strategy involves the yearly administration of four doses of sulfadoxine–pyrimethamine–amodiaquine (SPAQ) to children aged 3–59 months during high malaria transmission seasons ^9, 10^. Chemotherapy, and particularly chemoprevention, remains one of the key strategies in the control and management of the disease in children. However, these strategies have suffered setbacks as a result of the emergence and spread of genotypes of *Plasmodium falciparum* (Pf) resistant to available antimalarial drugs ^11, 12^. This resistance to antimalaria presents a major challenge to the elimination and eradication of the disease ^13^. In highly vulnerable groups like pregnant women and children under the age of five years, it is used to prevent infection ^14– 16^. In Cameroon, resistance to many antimalarial drugs, like chloroquine (CQ), sulfadoxine–pyrimethamine (SP), and amodiaquine, has been reported ^17^. The selection pressure of drugs delivered as monotherapies drives the emergence and spread of antimalarial drug resistance in Cameroon. The emergence and spread of chloroquine resistance in Cameroon saw its replacement with amodiaquine as a first line treatment in 2002 and SP as a temporary measure prior to the adoption of the WHO’s recommendation for artemisinin-based combination therapies (ACTs) ^18, 19^. But SP is still used for intermittent preventive treatment in pregnant (IPTp) women ^20^. Sulfadoxine–pyrimethamine (SP) in combination with amodiaquine (AQ) is used for seasonal malaria chemoprevention (SMC). As a measure to detect and track the emergence and spread of resistance to this important combination, molecular characterization of the *P. falciparum* dihydrofolate reductase ( *DHFR*) and *P. falciparum* dihydropteroate synthase genes ( *DHPS*) are undertaken in areas where the drug combination is used for chemoprevention ^21^. SP inhibits the activities of DHFR and DHPS enzymes in the parasite folate synthesis pathway, depriving the parasite of essential cofactors required for the synthesis of building blocks of nucleic acids essential for replication and transcription ^22^. Resistance to pyrimethamine is mediated by point mutations in *DHFR* located in chromosome 4, while sulfadoxine resistance is conferred by non-synonymous mutations in *DHPS* located in chromosome 8 in the *P. falciparum* genome. The following amino-acid changes occurring in a stepwise fashion in DHFR (namely N51I, C59R, and S108N, also known as the DHFR triple-mutant) have been associated with reduced susceptibility to pyrimethamine, with the single nucleotide polymorphism at codon 108 a strong predictor for the occurrence of the DHFR triple-mutant. In addition, the 164L mutation common in Southeast Asia has been shown to confer high grade SP resistance ^22– 24^. This mutation is rare in Africa but was found in a cluster of patients in southwest Uganda ^25^, where it occurs independently in the background of the triple DHFR (N51I, C59R, +S108N) or the double mutant (N51I+ S108N). Five mutations in DHPS at codons S436A, A437G, K540E, A581G, and A613S/T have been implicated in sulfadoxine resistance, with mutations at codons A437G, K540E, and A581G being the core mutations. Several studies have shown that, in Africa, the combination of the quintuple mutant IRNGE (N51I, C59R, and S108N and A437G and K540E) is strongly associated with resistance to SP ^15, 16, 24^, especially in East Africa. The mutations occur in a stepwise fashion. Parasites harboring mutation at codon 581 of DHPS have emerged to be associated with a reduced effectiveness of SP used for intermittent preventive therapy in pregnancy ^26^. A change in policy should be envisaged when the prevalence of the DHPS mutation at codon 581 is > 10% in a background of > 95% of the quintuple IRNGE mutation ^27^. The WHO recommends SMC only in areas where the prevalence of DHPS 540E mutation is < 50% ^28^. With the withdrawal of SP for *P. falciparum* treatment, the prevalence of single nucleotide polymorphisms in DHFR and DHPS have decreased in some countries ^29, 30^. By contrast, the prevalence has increased in other countries, raising questions whether withdrawal would lead to a return of wild-type parasites (as has been the case with the withdrawal of chloroquine) ^31^. Factors including drug target, nature of genes, drug pressure, and host/parasite genetic background may influence the persistence of resistance conferring single nucleotide polymorphisms differently after drug withdrawal from use. As a consequence, to ensure the prophylactic efficacy of SMC and to support the National Malaria Control Program (NMCP)’s policies, the present study was carried out to determine SP resistance genotypes of *P. falciparum dhfr* and *dhps* in 2016 to start and in 2020 after five rounds of SPAQ administration in SMC.

## Methods

### Study site

The Far North Region of Cameroon (9°40′ N–13°05′ N and 12°15′ E–16°45′ E) is a Sudano–Sahelian zone covered by savannah and steppes. It has a total surface land area of 34,263 km ^2^ and is bordered to the south by the North Region, to the east by Chad, and to the west by Nigeria. The climate is semi-arid with a single rainy season; most of the year is hot and dry. The Far North Region’s capital is Maroua, which lies along the same belt stretching from the West African country of Gambia through Burkina Faso and Niger to Northern Nigeria and across Tchad and towards Sudan. This region experiences high precipitation during the rainy season, and therefore is prone to malaria epidemics ^8^.

The North Region has a surface area of about 66,090 km2, representing almost half of northern Cameroon. It is bordered to the north by the Far North Region, to the south by the Adamawa Region, to the west by Nigeria, to the east by Chad, and to the south east by the Central African Republic. Garoua is the capital of the North Region, with coordinates 60°24′ N and 100°46′ E. The vegetation here is of Guinea–savanna type, with an annual average rainfall of 380 mm with approximately 4 months of rainfall. This region is home to many ethnic groups, with agriculture as the prominent activity of the region ^8^.

In Cameroon, SMC with SPAQ has been implemented in the North Region and the Far North Region of Cameroon since 2016. Its introduction was widescale, exposing over 1.5 million children aged 3–59 months to SPAQ, yearly raising concerns regarding the development of resistance. These northern regions are suitable environments for the establishment of SMC, as they experience seasonality in the transmission of malaria. This transmission occurs during the rainy season ^5^.

### Ethical consideration

The study proposal was reviewed and approved by the Cameroonian National Ethics Committee for Human Health Research (No. 2015/03/567/CE/CNERSH/SP and No. 2018/01/961/CE/CNERSH/SP). Study authorizations were obtained from the Ministry of Public Health and from the District Hospitals, where the study was conducted. All consent-related documents were made available in French and English. The study objectives, sampling techniques, benefits, and risks of being part of the study were explained to the parents and guardians of the children. Those who consented signed the consent form. Guidelines for good clinical laboratory practice were implemented in the laboratory for molecular analysis.

### Study design

The targeted population was children aged between 6 and 120 months. Samples was collected from May to June in 2016 and from October to November in 2020. Children who came for consultation at the outpatient department of the hospital were enrolled based on an axillary temperature ≥ 37.5 °C or with a history of fever in the past 24 h, no recent history of antimalarial drug intake during the previous week, parental authorization and assent signed, and diagnosed positive for malaria by a rapid diagnostic test (SD Bioline ^®^ HRP-2 Pf) (Pan, Abbot Diagnostics Inc, Giheug-gu, Republic of Korea) and confirmed by microscopy using Giemsa-stained blood smears (The Giemsa was gotten from Atom scientific Ltd, United Kingdom, Cat N ^o^ 122611)

Finger-prick blood was collected from all children and used for the parasitological confirmation of malaria. Dried blood spots were prepared on Whatman 3 MM Whatman filter papers from children who tested positive for malaria. The filter papers were air-dried, packaged individually in zip lock bags with desiccant, and transported to the Laboratory of Public Health Research Biotechnologies of the University of Yaounde 1, where genotyping analysis was performed.

## DNA extraction

The parasitic DNA was extracted from the dried blood spots using the Chelex®-100 (Bio-Rad laboratories, SIGMA) (Inc. Marnes-la-Coquette, France) method, as previously described ^32^. Briefly, dried blood spots were isolated from filter papers with sterile scissors and placed in 0.5 % saponine prepared in Phosphate Buffered Saline (PBS) overnight at +4 °C. The following day, it was washed with 1× PBS and incubated for 30 min at +4 °C. The brown solution was discarded and the filter paper transferred to a 20% hot (100 °C) Chelex solution in DNAse free water followed by vigorous vortexing at 5 min intervals. The tubes were centrifuged at 10,000× *g* for two minutes and the supernatant transferred to a new tube, centrifuged again, and the last transfer was made to an Eppendorf tube that was labeled with the study code. The extract was stored at −20 °C until use.

### DNA amplification and restriction enzyme digestion

The *P. falciparum dhfr* and *dhps* were amplified using nested PCR. Specific primers targeting the gene fragments of interest and other master mix constituent were obtained from Inqaba Biotech (Pretoria, South Africa) and used according to the previously described methods with modification ^33, 34^. The reaction mixture for the first amplification included 18.25 μL of nuclease free water, 2.5 μL of 10× thermopol buffer, 0.5 μL of 10 mM dNTPs (dATP, dGTP, dCTP, dTTP), 0.25 μL of 2.5 μM of each primer, 0.25 μL of Taq DNA polymerase (5 units/μL), and 3 μL of parasite DNA extract, giving a total of 25 μL. In the second amplification reaction, the reaction mixture included 20.25 μL of nuclease free water, 2.5 μL of 10× thermopol buffer, 0.5 μL of 10 mM dNTPs (dATP, dGTP, dCTP, dTTP), 0.25 μL of 2.5 μM of each primer, 0.25 μL of Taq DNA polymerase (5 units/μL), and 1μL of amplicon of the first amplification reaction, giving a total of 25 μL.

The tubes were subsequently subjected to the following reaction conditions in the thermocycler Biometra T3, UK. For *dhfr* outer PCR reaction, a pre-denaturation at 94 °C for 3 min, 40 cycles of an initial denaturation at 94 °C for 1 min, primer hybridization at 50 °C for 2 min, extension at 72 °C for 2 min, followed by a final extension at 72 °C for 10 min. For the *dhfr* nested reaction, a pre-denaturation at 94 °C for 2 min, 35 cycles each of an initial denaturation at 94 °C for 1 min, primer hybridization at 45 °C for 1 min, extension at 72 °C for 2 min, followed by a final extension at 72 °C for 10 min. The same amplification program for the outer and nested for *dhfr* was used for *dhps,* except that the pre-denaturation step was 3 min for the nested reaction. The number of amplification cycles for the nested was 35.

The PCR amplification products were then analyzed on a 2% agarose gel and visualized under the UV transilluminator for verification of the expected band size for each of the amplicons prior to restriction digestion. The PCR amplicons of the second PCR amplification were digested with site-specific restriction enzymes. The following restriction enzymes were used: for *dhfr*, TSP5091, Xmn I, and Alu 1 to identify N51I, C59R, and S108N mutations, respectively; for *dhps*, AvaII and FokI to identify A437G and K540E mutations, respectively ( Table 1). The restriction enzymes was gotten from New England Biolab. The 3D7 strain parasites (obtained from the London School of Hygiene and Tropical Medicine courtesy of Dr Khalid Beshir) were used as a positive control in the experiments. A no-template control was used as the negative control.

**Table 1.** Primer sequences, PCR reaction conditions, and restriction enzymes used to genotype samples in *dhfr* and *dhps* in samples collected in 2016 and 2020 in Northern Cameroon.

Genes	Mutation	Primers		PCR Conditions	Restriction Enzyme
DHFR Primary		M1	5’TTTATGATGGAACAAGTCTGC3’ 5’ AGTATATACATCGCTAACAGA3’	94 °C-3 min, 94 °C-1 min, 50 °C-2 min, 72 °C-2 min, ×40, 72 °C-10 min, 4 °C-hold	
		M5			
DHFR Nested	N51I	M3	5’TTTATGATGGAACAAGTCTGCGACGTT3’ 5’AAATTCTTGATAAACAACGGAACCTttTA3’	94 °C-2 min, 94 °C-1 min, 45 °C-1 min, 72 °C-2 min, ×35, 72 °C-10 min, 4 °C-hold	TSP5091
		F/			
	C59R	F	5’GAAATGTAATTCCCTAGATATGGAATATT3’ 5’TTAATTTCCCAAGTAAAACTATTAGAGCTTC3’	94 °C-2 min, 94 °C-1 min, 45 °C-1 min, 72 °C-2 min, ×35, 72 °C-10 min, 4 °C-hold	XmnI
		M4			
	S108N	F	5’GAAATGTAATTCCCTAGATATGGAATATT3’ 5’TTAATTTCCCAAGTAAAACTATTAGAGCTTC3’	94 °C-2 min, 94 °C-1 min, 45 °C-1 min, 72 °C-2 min, ×35, 72 °C-10 min, 4 °C-hold	Alu I
		M4			
DHPS Primary		R2	5’ AACCTAAACGTGCTGTTCAA3’ 5’ AATTGTGTGATTTGTCCACAA3’	94 °C-3 min, 94 °C-1 min, 50 °C-2 min, 72 °C-2 min, ×45, 72 °C-10 min, 4 °C-hold	
		R/			
DHPS Nested	A581G	L	5’ATAGGATACTATTTGATATTGGACCAGGATTCG3’ 5’TATTACAACATTTTGATCATTCGCGCAACCGG3’	94 °C-2 min, 94 °C-1 min, 45 °C-1 min, 72 °C-2 min, ×35, 72 °C-10 min, 4 °C-hold	BstUI
		L/			
	K540E	K	5’TGCTAGTGTTATAGATATAGGATGAGCATC3’ 5’CTATAACGAGGTATTGCATTTAATGCAAGAA3’	94 °C-2 min, 94 °C-1 min, 45 °C-1 min, 72 °C-2 min, ×35, 72 °C-10 min, 4 °C-hold	FokI
		K/			

The restriction digestions were carried out overnight as indicated by the manufacturer‘s protocol (New England Biolabs (UK) Ltd.) and as previously described ^23, 35, 36^. The restriction fragments were analyzed on a 2% agarose containing 0.5 μg/mL ethidium bromide and alongside a 100 bp molecular weight maker and visualized under a UV transilluminator.

### Data analysis

The prevalence of mutations in *dhfr* (I51, R59, and N108) and *dhps* (G437 and E540) were determined for each time point. In samples where both mutant and wild-type alleles were identified, they were scored as mixed infections and, subsequently, as pure mutants. R software (version 4.2.2, R Foundation for Statistical Computing, Vienna, Austria) [ https://cran.rstudio.com/bin/windows/contrib/4.2/] was used to conduct analysis. The primary outcome was the frequency of alleles and haplotypes in the two time points and the differences in frequency of individual alleles and haplotypes between the two time points. Chi-square (χ ^2^) was performed to evaluate the differences among the same group of antifolate resistance markers between the time points. A *p*-value ≤ 0.05 was considered statistically significant.

## Results

Over 405 children aged 6 to 120 months with acute uncomplicated malaria were recruited in 2016 and 2020. Of the 405 samples returned for analysis, 201/405 (49.63%) were collected in 2016 and 204/405 (50.37%) were collected in 2020 ( Table 2(a)) ^37^. Samples without correct labeling and with poorly spotted dried-blood spots were excluded from the analysis. Parasitic DNA was extracted from the returned 405 samples and quantified with a Nanodrop (Thermo Fisher Scientific, Carlsbad, CA, USA). Molecular assays were performed for three DHFR and two DHPS mutations commonly known to occur in Africa.

**Table 2.** ( **a**): Proportion of single nucleotide polymorphism on *Plasmodium falciparum* dihydropteroate synthase genes in 2016 and 2020. ( **b**): proportion of single nucleotide polymorphism on *Plasmodium falciparum* dihydropteroate synthase genes.

(a)									
Gene	Locus	Amino Acid	Genotype	Alleles	Y2016	Y2020	Total	X ^2^	*p*
*DHFR*	TCA	Ser	Wild-type	S1O8	0 (0.0%)	2 (1.4%)	2 (0.6%)	2.206	0.9
	AAT	Asn	Mutant	108N	172 (98.9%)	137 (96.5%)	309 (97.8%)		
	TCA/AAT	Ser/Asn	Mixed	S108N	2 (1.1%)	3 (2.1%)	5 (1.6%)		
	TGT	Cys	Wild-type	C59	8 (4.6%)	11 (7.7%)	19 (6.0%)	6.373	0.702
	CGT	Arg	Mutant	59R	163 (93.7%)	129 (90.8%)	292 (92.4%)		
	TGT/CGT	Cys/Arg	Mixed	C59R	3 (1.7%)	2 (1.4%)	5 (1.6%)		
	AAT	Asn	Wild-type	N51	43 (24.7%)	28 (19.7%)	71 (22.5%)	9.338	0.407
	ATT	Ile	Mutant	51I	126 (72.4%)	108 (76.1%)	134 (42.4%)		
	AAT/ATT	Asn/Ile	Mixed	N51I	5 (2.9%)	6 (4.2%)	11 (3.5%)		
					174 (55.1%)	142 (44.9%)	316/405		
(b)									
Gene	Locus	Amino Acid	Genotype	Alleles	Y2016	Y2020	Total	X ^2^	Pv
*DHPS*	GGT	Gly	Wild-type	G437	86 (48.1%)	66 (47.5%)	152 (47.8%)	6.305	0.709
	GCT	Ala	Mutant	437A	79 (44.1%)	61 (43.9%)	140 (44.0%)		
	GGT/GCT	Gly/Ala	Mixed	G437A	14 (7.8%)	12 (8.6%)	26 (8.2%)		
	AAG	Lys	Wild-type	K540	173 (96.6%)	135 (97.1%)	308 (96.9%)	1.769	0.940
	CAA	Glu	Mutant	540E	6 (3.4%)	2 (1.4%)	8 (5.5%)		
	AAG/CAA	Lys /Glu	Mixed	K540E	0 (0.0%)	2 (1.4%)	2 (0.6%)		
					179 (44.2%)	139 (34.3%)	318/405		

A = adenine, C = cytosine, G = guanine, pv = *p* value, Ala = A = alanine, Gly = G = glycine, Glu = E = glutamine, *pfDHPS* = *Plasmodium falciparum* dihydropteroate synthase, X ^2^ = chi square, CI = confidence interval, Y2016 = year 2016, Y2020 = year 2020.

Of the 201 samples in 2016, 174/201 were successfully genotyped for *dhfr* and 179/201 for *dhps*, giving a genotyping rate of 86.57% and 89.05%, respectively. Concerning the 2020 samples, 142/204 and 139/204 were successfully genotyped for *dhfr* and *dhps*, with a genotyping rate of 69.61% and 68.14%, respectively ( Table 2(a) and (b)). This drop-in genotyping rate could be due to the sampling technic used and the conservation of samples ^38^. Indeed, in 2016, samples were directly spotted on filter papers from the field, while, in 2020, samples where first collected in EDTA tubes before been spotted on filter papers. The overall genotyping rate for the samples collected throughout this study was 78.02% (316/405) for *dhfr* and 78.52% (318/405) for *dhps.*

The prevalence of five-point mutations for both genes was determined for both years. Three single nucleotide polymorphisms (SNPs) were genotyped for *dhfr*. The samples showed a high prevalence of mutations N51I, C59R, and S108N in *dhfr*, all known to be associated with pyrimethamine resistance. The mutation S108N was the most represented, with a 100% prevalence in 2016 and 98.6% in 2020 ( Table 2a). Two SNPs were genotyped in *dhps* at codons 437 and 540 of *dhps*. The two mutations were detected at variable frequencies for both years. The prevalence of the mutant genotype of 3.4% (6/179) in 2016 and % (4/139) in 2020 was observed at codon K540E following enzyme restriction analysis. Regarding codon A437G, a prevalence of the mutant genotype of 51.9% (93/179) and 52.5% (73/139) was observed in 2016 and 2020, respectively ( Table 2b).

Pearson’s chi square was used to test whether there was a difference in the frequency of distribution of the SNPs at different time points. No significant difference in the distribution of alleles was observed between 2016 and 2020 for both *dhfr* and *dhps,* as shown in Table 2(a) and (b).

**Figure 1.**
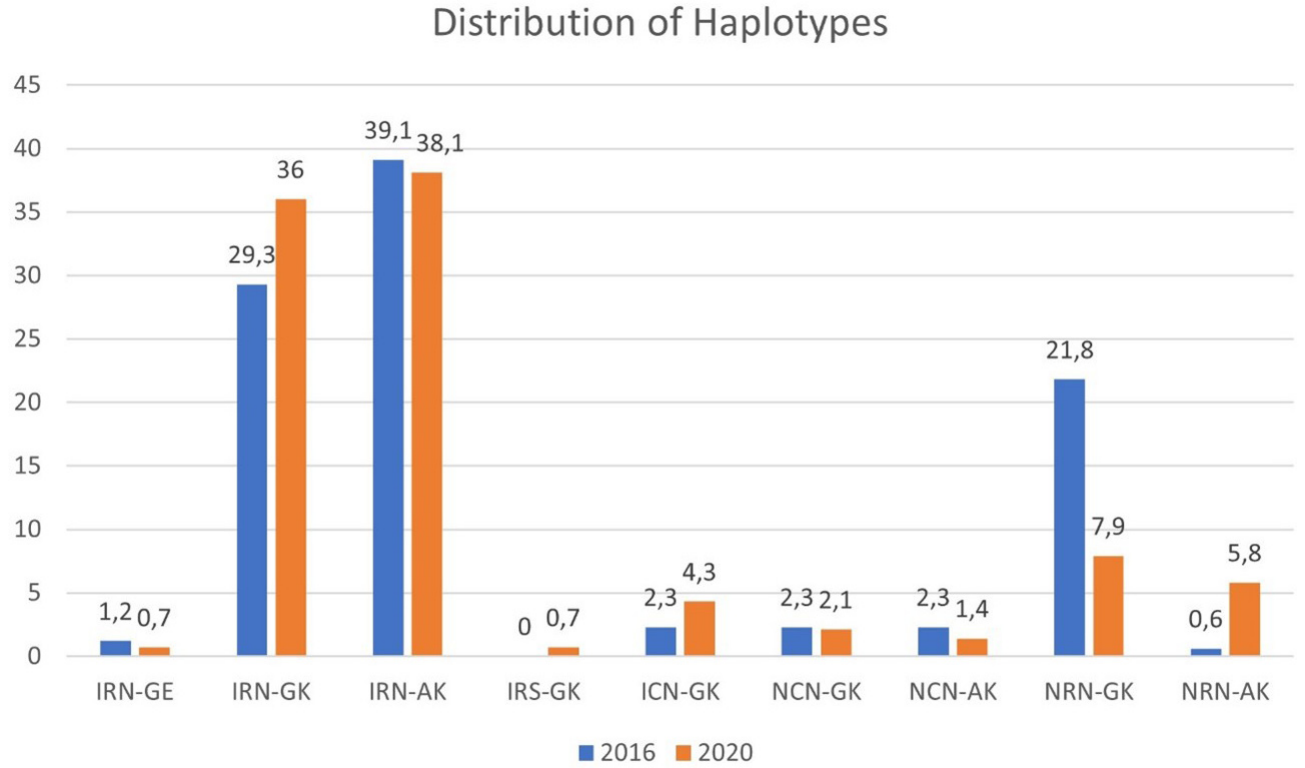
Proportions of haplotypes of *dhfr* and *dhps* resistance mutations observed in samples collected in the time points 2016 and 2020.

A comparison of the combined mutations in the DHPS and DHFR genes between 2016 and 2020 was undertaken, as shown in Table 3. Haplotypes including single, double, triple, quadruple, and quintuple mutations were constructed and included in the comparison between time points ( Figure 1). All mixed infections (presence of both mutant and wild-type genotype) were considered as mutants. The haplotype containing the quadruple mutation IRN-G was found in 51/174 (29.3%) in 2016 and 50/139 (36.0%) in 2020 ( *p* < 0.0001).

**Table 3.** Haplotype frequencies of the dihydropteroate synthase (DHPS) and dihydrofolate reductase (DHFR) resistance conferring mutations in Plasmodium falciparum by year of sampling.

	2016 n (%)	2020 n (%)	Total	P value
**IRN-GE**	2(1,2)	1(0,7)	3(1,0)	X ^2^= 56 ; P < 0.0001
**IRN-GK**	51(29,3)	50(36,0)	101 (34,5)	
**IRN-AK**	68(39,1)	53(38,1)	111(37,9)	
**IRS-GK**	0(0,0)	1(0,7)	1(0,3)	
**ICN-GK**	4(2,3)	6(4,3)	10(3,4)	
**NCN-GK**	2(2,3)	3(2,1)	5(1,7)	
**NCN-AK**	2(2,3)	2(1,4)	4(1,4)	
**NRN-GK**	38(21,8)	11(7,9)	49(16,7)	
**NRN-AK**	1(0,6)	8(5,8)	9(3,1)	

## Discussion

Routine surveillance of *P. falciparum* resistance is essential to monitor the efficacy of treatment and implementation of malaria chemoprophylactic guidelines. This study aimed at assessing the *P. falciparum* antifolate resistance markers in 2016 just before the implementation of seasonal malaria chemoprevention for malaria control and in 2020 after five rounds of the implementation in the northern regions of Cameroon.

For the two time points, single nucleotide polymorphisms (SNPs) in the DHFR (N51I, C59R, and S108N) and DHPS (A437G, K540E) genes and the prevalence of DHFR / DHPS combined mutant associated with pyrimethamine ^39^ and sulfadoxine ^40^ resistance were examined. Early studies have demonstrated that the combination of the triple (51I/59R/108N) mutant with the DHPS double mutant (437G/540E), often referred to as the quintuple mutant, is a predictor of clinical SP treatment failure ^41^.

The prevalence of the SP mutations was almost the same for the two time points, with almost a 100% mutant rate at codon 108 (S108N). This was found to be in line with another study conducted in Cameroon, in which an almost unpreventable increase in mutation at codon 108 was observed from 48% in 1994–1995, 71% in 1997–1998, and 93% in 2000–2001 ^11^. The triple mutation of S108N, N51I, and C59R was observed in 100%, 95.4%, and 75.3% of the 2016 isolates, respectively. Similarly, 98.6%, 92.2%, and 80.3% mutation rates were observed in the 2020 isolates. This was similar to other studies conducted in Angola and Tanzania, in which a proportion of 100%, 93%, and 57% was observed in the isolates ^42^. Another study from Uganda also reported a similar trend in mutation rate at codons S108N, N51I, and C59R ^43^. The same situation was also found by Mahamar et al. in 2022, who found similar trends with baseline samples compared with 3 years after SMC administration in Mali ^15^. These results align with other studies to suggest that mutations in *dhfr* have almost reached fixation ^44^ and may therefore have little to contribute further in the resistance phenotype ^15^ but may provide the genetic background for the expression of high-grade resistance

Generally, a low prevalence of isolates carrying the mutation K540E was observed. It was higher (3.4%) in the 2016 isolates compared with those obtained from the 2020 (2.8%) isolates. The low prevalence of this mutation suggests that the preventive treatment with SP should be reinforced in this area given its potential benefits in preventing malaria ^45^. This prevalence was similar to that of another study carried out by Mariangela et al. on samples collected in 2017 in two semi-urban cities of Cameroon. These results differ from some countries where SMC is being administered, with very low prevalence (2.3% in Guinea; 0.39% in Niger; 0.30% in Mali) to relatively no prevalence (0.0%) in Chad, Burkina Faso, and Senegal in samples collected in 2018 ^25^. In most of these countries, SMC is administered nationwide (Burkina Faso, Senegal, Mali) and has been implemented for more than 10 years ^12, 46, 47^. In countries neighboring Cameroon, this mutation prevalence was 6.25% in Gabon in 2007, 0.8% in Congo in 2004, 5.2% in the Central African Republic in 2004, 11% in the Sao Tome and Principe islands in 2004, 24% in Nigeria in 2004, and 3.2% in Equatorial Guinea in 2020. K540E has been observed to be higher in most East African countries and rare in West African countries ^25, 42, 48^. In Kenya, for instance, 91% mutations at codon K540E have been reported, reflecting an increase in *P. falciparum* resistance to SP since its withdrawal in Kenya ^24, 49^.

Analysis of the DHPS double mutation GE at positions 437 and 549 revealed a 0.01% (one isolate) proportion in the 2016 isolates and a 2.8% proportion of double mutation in the 2020 isolates. The double mutation at codon A437G and K540E of DHPS has been shown to be consistently associated with the risk of in vivo clinical failure ^50, 51^. Additional mutation(s) at positions beyond 437 and 540 notable at codons 581 and 613 are associated with decreased parasite sensitivity to the sulfa drugs ^24, 42, 52, 53^.

The present study revealed a high prevalence of the triple (IRN) and quadruple (IRNG) haplotype for either years or time points, with an increase in 2020 for the quadruple haplotype from 29.3% to 36.0% ( *p* < 0.0001). This study is in line with similar studies ^54^ that have demonstrated that about 75% of the *P falciparum* parasite carried triple mutations in *dhfr*. These haplotypes have also been demonstrated to be prevalent in Western and Central Africa ^25^ and are associated with reduced sensitivity to SP ^55, 56^. A similar pattern was found in areas where SMC has been administered ^57, 58^ over the years. This result, however, is different from a previous observation in Thailand, where the prevalence in point mutations was found to transition from double through triple to quadruple in the course of the years ^59– 61^. This difference may be justified by the fact that the cessation of SP for the treatment of malaria and its reorientation into intermittent preventive treatment in women and in seasonal malaria chemoprevention reduced pressure on the parasite ^41, 62^. However, the role of pressure from other sources such as from the use of trimethoprim and sulfamethoxazole for the treatment of widespread bacterial infections cannot be completely overlooked ^63^.

The quintuple haplotype IRNGE was found to be low in just three (0.9%) samples over the two timepoints: two (1.1%) in 2016 and one (0.7%) in 2020. This observation is not surprising because of the general finding that the K540E mutation is rare in West Africa ^12, 14, 64^. This is slightly high compared with other sites where SMC has been administered in which this quintuple mutation has not been found ^25^. This observation is opposed to observations in east and southern African regions such as Kenya and Mozambique, where quintuple SP resistance mutations are reported to be above 78%, while the triple mutant has reached fixation ^65^. By contrast, we observed an increase in the haplotype with quadruple mutations (IRN-G) between 2016 and 2020, which is more consistent with the role of this resistant haplotype and reduced treatment response in West Africa ^66^. Other mutations that have been frequently seen to occur alongside other established mutations may play a part in SP resistance in West Africa. In particular, the I431V mutation, which has recently been characterized in infections in intermittent preventive treatment in pregnancy programs ^48, 66, 67^, and more generally in the population ^68^, is beginning to gain attention regarding the putative role it may play in regional differences in parasite molecular responses to SP pressure. Future studies evaluating the role of this mutation, as well as others, may shed more light on the dynamics of drug resistance in chemoprevention programs in malaria-endemic countries of Africa and Cameroon in particular.

## Conclusions

Our findings show that, after five rounds of SMC implementation in Cameroon (2016–2020), there was no significant increase in known mutations associated with SP resistance in the study areas. However, the increase in the IRN-G resistance conferring haplotype highlights the importance of continuous monitoring of these markers and new putative resistance mutations with long-term implementation of SMC. This will enable timely decision making to optimize malaria chemoprevention in the long-term and contribute to the elimination of malaria.

## Ethics and consent

The study was conducted in accordance with the Declaration of Helsinki, and approved by the Institutional Review Board (or Ethics Committee) of Cameroonian National Ethics Committee for Human Health Research (No. 2015/03/567/CE/CNERSH/SP, 13th March 2015; No. 2018/01/961/CE/CNERSH/SP, 4th January 2018). Written informed consent was obtained from all participants’ parents/guardians prior to their involvement in the study.

## Data Availability

Zenodo: Datasets for the resistance analysis before (2016) and after (2020) SMC in the North region of Cameroon. https://zenodo.org/doi/10.5281/zenodo.11536799 ^37^. Data are available under the terms of the Creative Commons Attribution 4.0 International license (CC-BY 4.0).
