# Supplementary material for: Plasmodium falciparum dhps and dhfr markers of resistance to sulfadoxine–pyrimethamine five years (2016–2020) after the implementation of seasonal malaria chemoprevention in Cameroon
Source: Wellcome Open Res. 2024 Jun 20;9:323. [Version 1] doi: 10.12688/wellcomeopenres.22347.1 (PMC11624434; doi:10.12688/wellcomeopenres.22347.1)
Supplement: Peer review history

# Reviewer report: Reviewer response for version 1

**DOI:** 10.21956/wellcomeopenres.24624.r97914

**Published:** 2024-12-06

**Authors:** Pedro Berzosa

**Affiliations:** 1 CIBERINFEC - CIBER Infectious Diseases (ISCIII), Institute of Health Carlos III, Madrid, Spain

I think the work is fine. It is something necessary after several rounds of SPAQ use, to know the status of the molecular resistance of *P. falciparum*.

**I have several doubts:**

 1) Why did they use RFLPs...I understand that sequencing is more expensive, but the possibility of knowing SNPs by sequencing is better .

 2) On the other hand, since the SMC is SPAQ, they have studied only SNPs in *pfdhfr *and *pfdhps*, why they have not included genes like *pfmdr1 *and *pfcrt*? mutations in these genes can help to see the state of resistance to AQ.

 Have you considered doing so in the future, even with other samples?

 I think it is a good job. I work in Equatorial Guinea with the National Malaria Program and knowing the status of resistance in the neighboring country is very good for us.

Is the work clearly and accurately presented and does it cite the current literature?

Yes

If applicable, is the statistical analysis and its interpretation appropriate?

Yes

Are all the source data underlying the results available to ensure full reproducibility?

Yes

Is the study design appropriate and is the work technically sound?

Yes

Are the conclusions drawn adequately supported by the results?

Yes

Are sufficient details of methods and analysis provided to allow replication by others?

Yes

Reviewer Expertise:

I am head of the malaria and NTDs laboratory. I work on the line of antimalarial drugs resistance and genetic variability of P. falciparum, as well as in the line of study of the pfhrp2 and pfhrp3 genes and their relationship with false negatives in the use of malaria rapid tests. I have been collaborating for many years with the National Malaria Program of Equatorial Guinea.

I confirm that I have read this submission and believe that I have an appropriate level of expertise to confirm that it is of an acceptable scientific standard.

# Reviewer report: Reviewer response for version 1

**DOI:** 10.21956/wellcomeopenres.24624.r97922

**Published:** 2024-09-18

**Authors:** Abebe A Fola

**Affiliations:** 1 Purdue University, Brown University, Providence, RI, USA

**Reviewer Report**

**Title**: Plasmodium falciparum dhps and dhfr markers of resistance to sulfadoxine–pyrimethamine five years (2016–2020) after the implementation of seasonal malaria chemoprevention in Cameroon

**Overall Evaluation:**

 This manuscript by ChemCam and colleagues provides a timely and valuable examination of Plasmodium falciparum resistance to sulfadoxine–pyrimethamine (SP) before and five years after the implementation of seasonal malaria chemoprevention (SMC) in Cameroon. The study contributes important insights into the impact of SMC on drug resistance dynamics.

 However, several critical aspects need to be addressed to enhance the manuscript:

**Methods:**

 Site Selection and Malaria Transmission Intensity: The manuscript should clarify the criteria for selecting the study sites and provide details on malaria transmission intensity at these locations. Understanding transmission intensity is crucial as it influences drug pressure and resistance patterns.

 PCR Artifacts: The authors should describe the measures taken to control potential PCR artifacts during SNP calling to ensure the reliability of the resistance markers.

**Statistics:**

 Resistance Conferring Haplotype IRNG: The manuscript notes a rise in the IRNG haplotype in 2020. A detailed interpretation of this trend is needed, including its implications for resistance and the potential impact on treatment efficacy.

**Additional Data:**

 New Variants: The study should include information on any new variants that emerged after the implementation of SMC and their frequencies.

  Other Mutations: In addition to the IRN-GE haplotype, mutations such as I431V, A581G, and A613S/T should be examined in detail to provide a comprehensive overview of resistance markers.

**Discussion:**

 Regional Variations: The discussion should compare SP resistance markers in Western versus Eastern Africa. This comparison will help determine if different guidelines or strategies might be needed for different regions, as recommended by the WHO.

**Abstract**

 The abstract should be revised to incorporate these additional details and provide a clearer summary of the key findings, including the rise of the IRNG haplotype, the presence of new variants, and the regional variations in SP resistance markers.

 Overall, addressing these points will improve the clarity and impact of the study, offering a more comprehensive understanding of SP resistance in the context of SMC implementation.

Is the work clearly and accurately presented and does it cite the current literature?

Yes

If applicable, is the statistical analysis and its interpretation appropriate?

Partly

Are all the source data underlying the results available to ensure full reproducibility?

Partly

Is the study design appropriate and is the work technically sound?

Yes

Are the conclusions drawn adequately supported by the results?

Partly

Are sufficient details of methods and analysis provided to allow replication by others?

Partly

Reviewer Expertise:

Genomic epidemiology malaria focusing drug and diagnostic resistance

I confirm that I have read this submission and believe that I have an appropriate level of expertise to confirm that it is of an acceptable scientific standard.

# Reviewer report: Reviewer response for version 1

**DOI:** 10.21956/wellcomeopenres.24624.r91933

**Published:** 2024-08-14

**Authors:** Lamine Mahaman Moustapha

**Affiliations:** 1 Faculty of Science and Technology, Department of Biological Sciences, Université André Salifou, Zinder, Zinder, Niger

**Full report on the article: *Plasmodium falciparum* dhps and dhfr markers of resistance to sulfadoxine–pyrimethamine five years (2016–2020) after the implementation of seasonal malaria chemoprevention in Cameroon**

**Summary of the article:**

 The article investigates the prevalence of mutations in the *Plasmodium falciparum* dihydropteroate synthase (dhps) and dihydrofolate reductase (dhfr) genes, which are markers of resistance to sulfadoxine-pyrimethamine (SP), following the implementation of seasonal malaria chemoprevention (SMC) in Cameroon between 2016 and 2020. The study's objective is to evaluate the impact of SMC on the selection of these resistance markers over time.

**General comments:**

- Scientific Nomenclature: The term “ *Plasmodium falciparum*” should be corrected throughout the document to reflect proper scientific nomenclature. Specifically, the genus name should be capitalized, and the species name should be italicized (e.g., *Plasmodium falciparum*). This correction should be applied consistently in the manuscript.

**Study Site:**

- Description of study sites: The current description of the study sites lacks clarity. It is essential to provide a more explicit description of the specific locations where sample collection took place. Furthermore, I strongly recommend including a map to visually represent these sites, which will significantly enhance readers' understanding of the geographical context of the study.

**Study design:**

- Age Group Selection: The rationale behind choosing the age range of 6-120 months requires further explanation. Considering that the objective of the study is to determine the prevalence of SP resistance markers before and after SMC, it is important to clarify why this age range extends beyond the recommended SMC age group of 3-59 months. Additionally, since SP is also used in intermittent preventive treatment for pregnant women (IPTp), a detailed justification for the selected age group is necessary.
- Timing of sample collection: It is crucial to provide details about the specific months during which sample collection was conducted in 2016 and 2020. This information will help readers understand whether the samples were collected during, before, or after the SMC campaign cycles. Including these details will improve the transparency and comprehensibility of the study design.
- SMC administration evidence: Please specify whether you have any documented evidence that the children sampled had received the various SMC cycles. This information is vital to strengthen the study's conclusions.
- Sample size calculation: The manuscript would benefit from including details on how the sample size was calculated. This information is essential for assessing the robustness of the study's findings.

**DNA amplification and restriction enzyme digestion:**

- PCR Analysis: Please specify whether ethidium bromide was added, and if so, the quantity used. Additionally, details regarding the migration time, voltage, and amperage applied during electrophoresis should be included.
- Visual Representation: I recommend providing images of the agarose gel electrophoresis results to visually support the DNA amplification process described. This addition will enhance the credibility and reproducibility of your work.

**Results:**

- Prevalence Reporting: The sentence "The prevalence of the mutant genotype of 3.4% (6/179) in 2016 and % (4/139) in 2020 was observed at codon K540E following enzyme restriction analysis" is incomplete. Please correct this sentence by including the percentage prevalence for 2020.
- Quintuple Mutation Reporting: It is important to explicitly mention in the results section that the IRN-GE quintuple mutation was identified, even if its frequency was low. This finding is significant and should be highlighted in the text.

**Discussion:**

- Reference Appropriateness: The sentence "For the two-time points, single nucleotide polymorphisms (SNPs) in the DHFR (N51I, C59R, and S108N) and DHPS (A437G, K540E) genes and the prevalence of DHFR / DHPS combined mutant associated with pyrimethamine ^39^ and sulfadoxine ^40^" includes a reference (40) that appears to be incorrect. Please replace it with a more appropriate reference that specifically demonstrates the involvement of DHPS in sulfadoxine resistance.
- Typographical Error: In the sentence "Additional mutation(s) at positions beyond 437 and 540 notable at codons 581 and 613 are associated with decreased parasite sensitivity to the sulfa drugs ^24,42,52,53^," the word “sufa” should be corrected to “sulfa.”
- Study Limitations: I suggest adding one or two paragraphs discussing the limitations of your study. Addressing potential limitations will provide a more balanced perspective and help readers better understand the context of your findings.

**Conclusion:**

 The authors have conducted a valuable study on the prevalence of SP resistance markers in *Plasmodium falciparum* over a critical period following the implementation of SMC in Cameroon. However, the manuscript would greatly benefit from the revisions suggested above to enhance its clarity, scientific soundness, and overall impact.

 These revisions should be addressed to ensure that the article is scientifically robust and provides clear and actionable insights for the readers.

Is the work clearly and accurately presented and does it cite the current literature?

Yes

If applicable, is the statistical analysis and its interpretation appropriate?

Yes

Are all the source data underlying the results available to ensure full reproducibility?

Yes

Is the study design appropriate and is the work technically sound?

Partly

Are the conclusions drawn adequately supported by the results?

Yes

Are sufficient details of methods and analysis provided to allow replication by others?

Partly

Reviewer Expertise:

Immunology of Infectious DiseasesParasitic DiseasesDrug ResistanceMolecular ParasitologyInfectious Disease Control and PreventionMedical ParasitologyImmunoparasitologyFalciparum MalariaPlasmodium FalciparumInfectious Disease SurveillanceInfectious Disease MedicinePCRPCR-RFLPSingle Nucleotide PolymorphismMolecular MarkersPoint MutationMalariaMolecular EpidemiologyMolecular DiagnosticsTropical DiseasesInfectious Disease EpidemiologyInfectious Disease TransmissionChemoprevention

I confirm that I have read this submission and believe that I have an appropriate level of expertise to confirm that it is of an acceptable scientific standard, however I have significant reservations, as outlined above.
